# Lung CD4+ Vα2.3+ T-cells in sarcoidosis cohorts with Löfgren’s syndrome

**DOI:** 10.1186/s12931-020-1327-0

**Published:** 2020-02-28

**Authors:** Pernilla Darlington, Susanna Kullberg, Anders Eklund, Johan Grunewald

**Affiliations:** 1 Respiratory Medicine Division, Department of Clinical Science and Education, Södersjukhuset and Karolinska Institutet, Stockholm, Sweden; 2 Respiratory Medicine Division, Department of Medicine Solna and Center for Molecular Medicine (CMM), Karolinska University Hospital and Karolinska Institutet, Stockholm, Sweden

**Keywords:** Diagnosis, Lung T-cell subsets, Vα2.3, Sarcoidosis

## Abstract

**Background:**

Sarcoidosis is diagnosed by a combination of typical clinical and radiological findings together with biopsy proof of non-caseating epithelioid cell granulomas in affected tissues and/or the cell distribution in bronchoalveolar lavage fluid (BALF). We aimed at investigating the usefulness of measuring the proportion of T-cell receptor (TCR) CD4+ Vα2.3+ T-cells in BALF as an additive marker to CD4/CD8-ratio to confirm the diagnosis.

**Methods:**

From a register consisting of 749 sarcoidosis patients [Löfgren’s syndrome (LS) *n* = 274, non-LS *n* = 475] with information on Vα2.3+ T-cells, an expansion of CD4+ Vα2.3+ T-cells (CD4+ Vα2.3+ T cells > 10.5% in BALF) was seen in 268 (36%). Controls were healthy volunteers (*n* = 69) and patients with other pulmonary conditions (*n* = 39), investigated because of suspicion of sarcoidosis.

**Results:**

A proportion of CD4+ Vα2.3+ T-cells in BALF > 10.5% was highly specific for sarcoidosis, with a specificity of 97% and with a sensitivity of 36% (*p* < 0.0001). Receiver operating characteristic (ROC) curves show that testing for CD4+ Vα2.3+ T-cells in BALF was a more useable test in individuals with LS [area under the curve (AUC) 0.82, *p* < 0.0001] compared to the whole patient group (AUC 0.64, *p* < 0.0001).

**Conclusion:**

In this study, we show that an increased proportion of CD4+ Vα2.3+ T-cells in BALF is highly specific for sarcoidosis. This suggests that this T-cell subset could be used as an additional tool to the CD4/CD8-ratio to support the sarcoidosis diagnosis, particularly in patients with LS but also in patients with non-LS.

## Background

An accumulation of CD4+ T-lymphocytes in bronchoalveolar lavage fluid (BALF), resulting in an increased CD4/CD8 ratio (> 3.5), has been shown to associate with sarcoidosis [1]. Further, an expansion in BALF of CD4+ T-cells expressing T-cell receptor (TCR) Vα2.3 gene segment has been identified in HLA-DRB1*03+ sarcoidosis patients, usually with Löfgren’s syndrome (LS) i.e. with an acute disease onset with bilateral ankle arthritis and/or erythema nodosum (EN), bilateral hilar lymphadenopathy (BHL), sometimes combined with parenchymal infiltrates and usually fever [2]. Dr. Sven Löfgren, described in his study from 1946 different causes of EN and where joint symptoms were reported in 149 of 185 patients, distributed fairly evenly over all etiological groups [3]. Within the cohort he found a group of patients with BHL, subsequently named to have “the bilateral hilar lymphoma syndrome”, today Löfgren’s syndrom [4]. Later, EN has been reported to be significantly more common in women with sarcoidosis [5], whereas joint symptoms without EN is seen preferentially in men [6].

In sarcoidosis today, bronchoalveolar lavage (BAL) is commonly used as a diagnostic method and in this study we aimed at investigating the usefulness of measuring the proportion of CD4+ Vα2.3+ T-cells, as an additional diagnostic test to the CD4/CD8-ratio, by evaluating the sensitivity and specificity for sarcoidosis of such BALF expansions.

## Methods

### Study subjects

This report is based on a register of 749 sarcoidosis patients who had been investigated with bronchoscopy and BAL for diagnostic purposes at the onset of the disease, and where the proportion CD4+ Vα2.3+ T-cells had been analyzed. A T-cell expansion was defined as a value higher than three times the median value of CD4+ Vα2.3+ T-cells in peripheral blood of healthy subjects as previously described [7]. The patients came from one center in Stockholm, Sweden, where they were diagnosed with sarcoidosis through typical clinical (e.g. fever, cough, EN, ankle arthritis/tendovaginitis) and radiographic manifestations, findings at bronchoscopy with BAL including an elevated CD4/CD8-ratio (> 3.5) and/or positive biopsies, in accordance with the criteria of the World Association of Sarcoidosis and other Granulomatous Disorders [8]. Chest radiographs in patients with sarcoidosis were classified into five stages: Stage 0 - normal; Stage I - bilateral hilar lymphadenopathy; Stage II - bilateral lymphadenopathy with parenchymal infiltrates; Stage III – parenchymal infiltrates alone; Stage IV - fibrotic bands and volume reduction [9]. Patients were defined as ever smokers if they had previously smoked or were current smokers.

Two hundred seventy-four out of the 749 patients had LS, see Table 1. The proportion of never-smokers in patients with LS in different radiographic stages were: 55% in stage I and 48% in stage II (there were missing information in two patients). The proportion of never-smokers among non-LS patients in different radiographic stages were: 38% in stage 0, 48% in stage I, 56% in stage II, 52% in stage III and 57% in stage IV (there were missing information in three individuals). For comparisons we used BAL cells from a control group (*n* = 108) including healthy volunteers (*n* = 69), patients investigated in a similar manner as the sarcoidosis patients but who turned out to have other diagnoses such as extrinsic allergic alveolitis (*n* = 7) and nonspecific interstitial pneumonia NSIP (*n* = 27), lymphoma (*n* = 1), other malignancies mimicking sarcoidosis (*n* = 2), cryptogenic organizing pneumonia (COP, *n* = 1) and Crohn’s disease (*n* = 1). All controls had chest X-rays performed. Patients with NSIP had in general an impaired lung function with a mean total lung capacity (TLC) of 69%, range 48–101% (there were missing information in 6 individuals), and 15 (56%) of them were never-smokers. Most had idiopathic NSIP, but in 8 cases there was an underlying inflammatory disease; three had dermatomyositis, and one respectively had rheumatoid arthritis, psoriasis, scleroderma, Sjögren’s syndrome and microscopic polyangiitis. Written informed consent was obtained from all subjects, and approval was granted from the regional ethical review board.

**Table 1:** Clinical characteristics of controls and patients

	Healthy controls	Non- sarcoidosis	LS	Non-LS
Subjects (n)	69	39	274	475
Gender M/F (n)	30/39	21/18	164/110	320/155
Age, years^a^	24 (18–56)	56 (23–78)	39 (21–72)	46 (24–78)
Never-smokers (n)	69	24	144	251
Lymphocytes^b^	6.0 (0.4–28.4)	22.5 (1.5–86.2)	23.9 (1.0–76.6)	26.7 (1.2–85.7)
CD4/CD8-ratio^a^	1.7 (0.3–6.4)	1.4 (0.2–3.2)	9.3 (0.4–56.8)	6.9 (0.4–46.0)
V-alfa 2.3^b^	4.5 (1.6–18.9)	5.3 (1.0–26.0)	21.2 (0.3–50.0)	6.4 (0.2–44.3)

^a^Values are median (min-max). ^b^ Values are percentages of bronchoalveolar lavage cells (min-max). *LS* Löfgren’s syndrome. In 26 sarcoidosis patients there were no data about CD4/CD8-ratio. There were no data regarding smoking history in 2 LS patients and 3 non-LS patients

### Sample processing

**Fig. 1:**
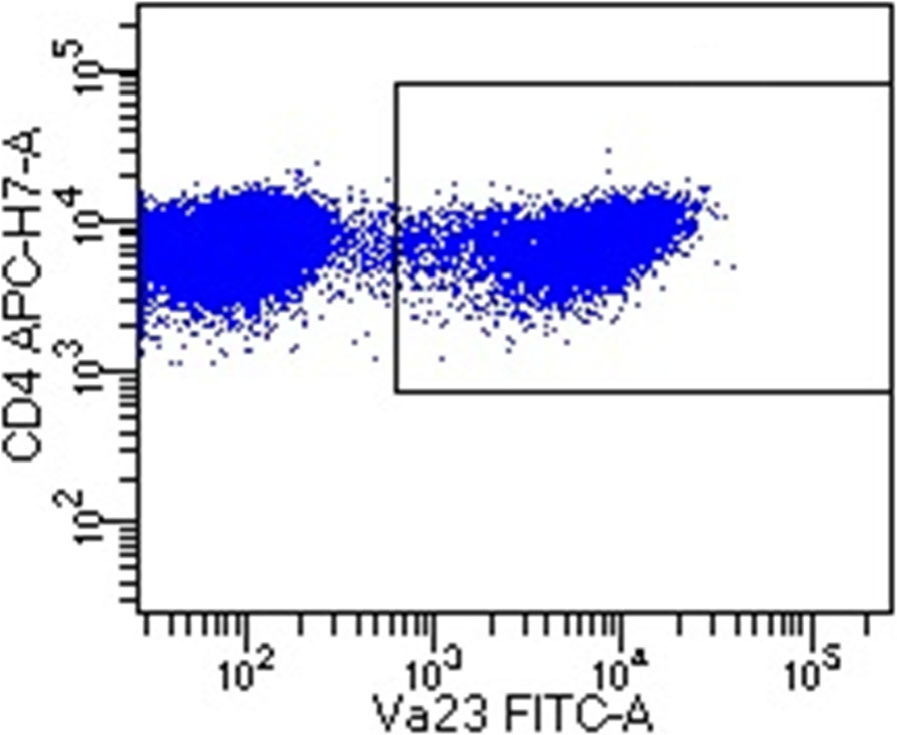
An example showing a population of CD4+ Vα2.3+ T-cells in BALF

The BAL was performed according to procedures earlier described [10]. The BALF was strained through a Dacron net (Millipore, Cork, Ireland) and centrifuged, thereafter the supernatants were removed. The cell pellet was resuspended in PBS and antibodies used as surface markers were added, and thereafter incubated at 4^o^ C for 20 min. After incubation, cells were washed twice with cell wash (BD Bioscience, Mountain View, CA, U.S.A.). Surface markers expressed on T cells were analyzed with flow cytometry using FACS CANTO II flow cytometer (BD Bioscience). Data were processed using a FACS Diva 6.1.2 software (BD Bioscience). The antibody used as surface marker for CD4+ Vα2.3+ T-cells was Vα2.3 (Thermo Scientific), see Fig. 1 for a typical staining.

### Statistical analysis

Sensitivity and specificity for using CD4+ Vα2.3+ T-cells as a diagnostic test was calculated considering patients and controls with CD4+ Vα2.3+ T-cells > 10.5% in BALF with diagnosed sarcoidosis as true positive and false positive if there was no evidence for the disease. Similar calculations were performed for the CD4/CD8-ratio, considering a ratio > 3.5 as true positive if sarcoidosis disease. Receiver operating characteristic (ROC) curves were calculated with percentage of CD4+ Vα2.3+ T-cells in controls in comparison with patients. Correlation was calculated with CD4/CD8-ratio on the x-axis and percentage of CD4+ Vα2.3+ T-cells on the y-axis.

## Results

Out of 749 patients with sarcoidosis, 268 subjects were identified with BALF CD4+ Vα2.3+ T-cells expansions. Out of these, 193 had LS. In contrast, all except three of the 108 controls had normal proportions of CD4+ Vα2.3+ T-cells in BALF, see Table 1. One of the individuals with a high expression in BALF was a healthy control without signs of sarcoidosis or any other pulmonary disease on chest X-ray, but with a CD4/CD8-ratio of 4.4. The other two were patients investigated because of suspicion of sarcoidosis with radiological NSIP, one with unknown cause and one with dermatomyositis, both with low CD4/CD8-ratio of 0.4 and 0.2.

A proportion of CD4+ Vα2.3+ T-cells in BALF > 10.5% was found to be highly specific for sarcoidosis, with a specificity of 97% but with a sensitivity of 36% (*p* < 0.0001), calculated on all 749 sarcoidosis patients, including LS as well as non-LS patients, and 108 controls. ROC curves show that testing for CD4+ Vα2.3+ T-cells in BALF was a more useable test in individuals with LS [area under the curve (AUC) 0.82, *p* < 0.0001] compared to the whole patient group (AUC 0.64, *p* < 0.0001).

**Fig. 2:**
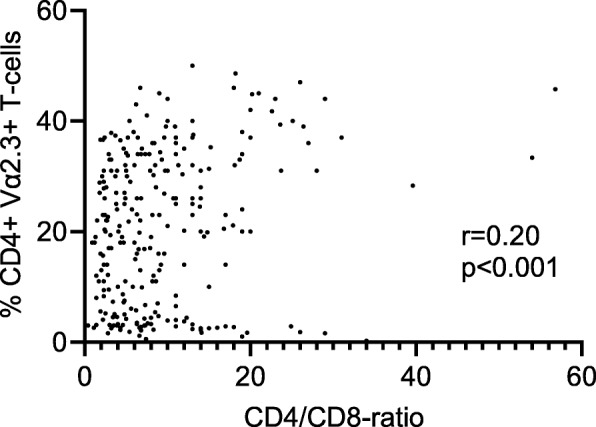
There was a positive correlation between the CD4/CD8-ratio and CD4+ Vα2.3+ T-cells in BALF in patients with Löfgren’s syndrome, *p* < 0.001 and *r* = 0.20

**Fig. 3:**
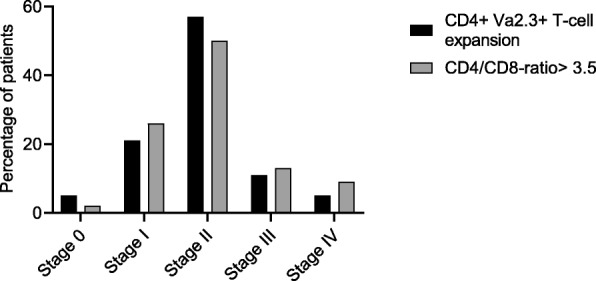
The proportions of non-LS patients with expansion of CD4+ Vα2.3+ T-cells in BALF (> 10.5%, *n* = 75) and elevated CD4/CD8-ratio (*n* = 325) respectively in different radiographic stages

For comparison, an elevated CD4/CD8-ratio was seen in 203 of the patients with LS, in 332 with non-LS and in 9 controls (all of them healthy volunteers). The specificity for CD4/CD8-ratio > 3.5 was 92% and the sensitivity 73% (*p* < 0.0001). ROC curves for the CD4/CD8-ratio showed a high diagnostic capacity in both the complete patient group with sarcoidosis (AUC 0.89, *p* < 0.0001) and in patients with LS (AUC 0.92, *p* < 0.0001). In patients with LS there was a positive correlation between the CD4/CD8-ratio and the percentage of CD4+ Vα2.3+ T-cells in BALF, see Fig. 2. The proportion of non-LS patients who had expansion of CD4+ Vα2.3+ T-cells in BALF (> 10.5%) or elevated CD4/CD8-ratio was distributed in a similar manner in different radiographic stages, see Fig. 3.

## Discussion

We set out to evaluate if an expansion of CD4+ Vα2.3+ T-cells per se in BALF could be used as an additional diagnostic tool for sarcoidosis. As shown, healthy controls and patients with other pulmonary diseases which constitute differential diagnosis to sarcoidosis only rarely seem to have such expansions. The analysis of this particular T-cell subset add value especially in sarcoidosis cohorts where Löfgren’s syndrome is frequently occurring and/or where HLA-DRB1*03 is common e.g. in Scandinavian countries. However, there were also patients with non-LS who had elevated levels of this specific subset of cells although less frequently. We suggest therefore that analyzing the proportion of CD4+ Vα2.3+ BAL T-cells in patients with sarcoidosis could be valuable as a complement to the CD4/CD8-ratio. A CD4/CD8-ratio > 3.5 is a more useful test as ROC-analysis show. However, increased CD4+ Vα2.3+ T-cells in BALF (> 10.5%) is highly specific for sarcoidosis (specificity 97%). As a result, in cases with normal or only marginally increased CD4/CD8-ratio but elevated CD4+ Vα2.3+ T-cells in BALF, further invasive procedures can be avoided.

A lung accumulation of CD4+ Vα2.3+ T-cells is well known and characteristic for sarcoidosis. The resulting increase in the CD4/CD8 ratio, in particular when > 3.5, has been suggested to be of help in diagnosing sarcoidosis [1]. The association between a CD4/CD8-ratio > 3.5 and sarcoidosis was also confirmed in this study, which to our knowledge is the largest patient cohort showing this. The results are consistent with a metanalysis of 999 patients and 886 controls [11]. A number of serological tests have also been proposed to reflect the activity and support the diagnosis of sarcoidosis. One of these is angiotensin-converting enzyme (ACE) which has been extensively investigated since it was first reported to be associated with sarcoidosis in 1975 by Lieberman [12]. The sensitivity was in a study including 101 patients diagnosed with sarcoidosis 62% and the specificity 76% [13]. However, ACE may be normal in early stages of the disease. Also, neopterin was found to be elevated in serum and to correlate with ACE [14] but for both substances the most important value seems to be when sequential analyses are made. According to a study on 232 sarcoidosis patients by Bargagli et al. [15] the enzyme chitotriosidase is also correlating to ACE and showed for sarcoidosis a high sensitivity (88.6%) and specificity (92.8%). In a study by Popevic et al. [16] the corresponding values for sensitivity were 82.5% and for specificity 70%. Serum-ACE in the latter study had 66% sensitivity and 54% specificity. Soluble IL-2 receptor has been shown by Bargagli et al. [17] to correlate to chitotriosidase and to be a useful marker of disease progression. Eurelings et al. [13] reported a sensitivity of 88 and 85% specificity in a total of 101 patients diagnosed with sarcoidosis. Several other attempts have been made to identify biomarkers. One of them is KL-6 that showed a sensitivity of 78% and seems to reflect the more severe disease stages well [18]. Another is serum amyloid A which is known to be involved in inflammatory reactions and immune disorders [19]. It has been reported to be increased in sarcoidosis and suggested to be valuable especially at follow-up. Taken together, no serological biomarker has so far proven ideal and in this setting we believe that CD4+ Vα2.3+ T-cells could provide additional valuable information in diagnosing and in some cases monitoring sarcoidosis. The Va2.3+ cells could perhaps be a component in the future of suggested multi-assays as proposed by Ramos-Casals et al. [20]. A weakness of the present study is the composition of the controls. We did not include patients with e.g. tuberculosis and only one with lymphoma since we seldom see patients with these diagnoses at the lung clinic. Studying the CD4+ T-cell subgroup that express Vα2.3, we found particularly in LS patients a strong association with the diagnose and a capacity to separate sarcoidosis from controls. In non-LS this finding was also existing but weaker.

The accumulation of CD4+ Vα2.3+ T-cells in the lung indicates the presence of specific lung-antigens, that are recognized by CD4+ Vα2.3+ T-cells, leading to a specific immune response in the lung. The function of these lung accumulated T-cells is unknown, although recent data show a preferential pairing with TCR Vb22, and a high degree of clonality, implicating these cells to interact with an as yet unknown antigen [21]. This is also in line with the finding that the percentage of CD4+ Vα2.3+ T-cells in BALF normalizes as the disease resolves [22]. The hypothesis has been further strengthened by the discovery of T-cells clones that are identical between different individual patients, as well as by a strong association with HLA-DRB1*03, which may be considered to present specific antigens for these T-cells [2]. The good prognosis in patients with CD4+ Va2.3+ T cells may indicate that precisely these T-cells for unknown reasons are particularly effective in their immune response to and elimination of such a potential antigen. Thus, the percentage of CD4+ Vα2.3+ T-cells in BALF may also serve as a prognostic marker as the proportion of patients with resolving disease increases gradually with increased proportions of CD4+ Vα2.3+ T-cells in BALF [23]. LS patients have the highest levels of CD4+ Vα2.3+ T-cells as well as the best prognosis and are in addition found to have very few extrapulmonary manifestations (EN and ankle arthritis excluded) [24].

How a presumed antigen is presented to the T-cells in sarcoidosis seems to play a role for the disease course. There is a correlation with HLA-DRB1*03 and subtypes of *13 and elevated levels of CD4+ Vα2.3+ T-cells in BALF [23]. Furthermore, the majority of patients with LS have either HLA-DRB1*03 and/or DRB3*01, in turn associated with good prognosis [25]. This phenomenon may explain why CD4+ Vα2.3+ T-cells is of best diagnostic use in LS patients. The other T-cell subsets that cause elevated CD4/CD8-ratio in patients with other HLA-types need to be investigated in further studies.

## Conclusion

In conclusion the results in this study indicate that Vα2.3 can be an additive diagnostic marker to the CD4/CD8-ratio for sarcoidosis. A low value does not exclude sarcoidosis, but an increased percentage of > 10.5% strengthens the diagnosis. In comparison, the CD4/CD8-ratio > 3.5 had a lower specificity for sarcoidosis but a higher sensitivity. Whether Vα2.3 may be of clinical value also in other populations than Scandinavian needs to be analyzed in further studies, as well as the significance in other granulomatous disorders.

## Data Availability

We have no specific “link” to any database, but data can be reproduced by buying commercial surface marker for CD4+ Vα2.3+ T-cells, Vα2.3 (Thermo Scientific) and then stain bronchoalveolar lavage fluid cells from patients and healthy, as we present in our manuscript.

## Abbreviations

ACE: Angiotensin-converting enzyme
AUC: Area under the curve
BAL: Bronchoalveolar lavage
BALF: Bronchoalveolar lavage fluid
BHL: Bilateral hilar lymphadenopathy
COP: Cryptogenic organizing pneumonia
EN: Erythema nodosum
LS: Löfgren’s syndrome
NSIP: Nonspecific interstitial pneumonia
ROC: Receiver operating characteristic
TLC: Total lung capacity
